# The Importance of Periodic Electrocardiograms in Individuals with Metabolic Disorders: A Policy Brief: Short title:Periodic ECG Monitoring in Metabolic Disorders

**DOI:** 10.31661/gmj.v15i.4206

**Published:** 2026-05-01

**Authors:** Seyed Rasool Nazemi Fard, Babak Pezeshki, Mojtaba Farjam

**Affiliations:** ^1^ Student Research Committee, Fasa University of Medical Sciences, Fasa, Iran; ^2^ Noncommunicable Diseases Research Center, Fasa University of Medical Sciences, Fasa, Iran

**Keywords:** Metabolic Disorders, Electrocardiography, QT Interval, Sudden Cardiac Death, Risk Assessment

## Abstract

Metabolic disorders, including obesity, type 2 diabetes, metabolic dysfunction-associated fatty liver disease (MAFLD), anemia, and insulin resistance, are increasingly prevalent and represent a major contributor to cardiovascular morbidity and mortality. Emerging evidence indicates that these conditions are associated with prolongation of the corrected QT (QTc) interval, a well-established marker of ventricular arrhythmias and sudden cardiac death. Individuals with metabolic disorders have a significantly higher risk of life-threatening arrhythmias, even in the absence of known cardiovascular disease. Insulin resistance, a central component of cardiometabolic dysfunction, has also been linked to QT interval prolongation. Despite this growing body of evidence, QTc monitoring is not currently included in routine clinical guidelines for the management of metabolic disorders. Electrocardiography (ECG) is a low-cost and widely accessible diagnostic tool that enables early identification of high-risk individuals and supports timely preventive interventions. Routine implementation of baseline and annual ECG-based QTc monitoring has the potential to reduce sudden cardiac death, improve cardiovascular risk stratification, and decrease healthcare costs associated with acute cardiac events. This policy brief advocates for the adoption of a national QTc monitoring strategy as a scalable and cost-effective approach to improving cardiovascular outcomes in patients with metabolic disorders.

## Executive Summary

Metabolic disorders including obesity, type 2 diabetes, metabolic dysfunction-associated fatty liver disease (MAFLD), anemia, and insulin resistance are highly prevalent and contribute substantially to cardiovascular morbidity and mortality. These conditions are characterized by disruptions in metabolic homeostasis affecting glucose, lipid, and energy metabolism [1].

Evidence from large Iranian cohort studies (Fasa and Isfahan) demonstrates a strong association between metabolic disorders and prolongation of the corrected QT (QTc) interval, which increases the risk of ventricular arrhythmias and sudden cardiac death [2][3]. This association is further supported by meta-analytic evidence [6]. Despite this, QTc monitoring is not included in current national guidelines for metabolic disease management.

This policy brief recommends the implementation of a national QTc monitoring program, including baseline and annual electrocardiogram (ECG) screening, physician training, and insurance coverage.

## Problem Statement

Metabolic disorders are increasing and place a large proportion of adults at risk for cardiovascular disease. Data from Iranian cohort studies show that these conditions increase the risk of life-threatening arrhythmias and sudden cardiac death by approximately 1.4-1.6-fold [4][5], even in individuals without prior cardiovascular disease.

Prolongation of the QT interval is associated with polymorphic ventricular tachyarrhythmias, which can rapidly lead to sudden cardiac death. In patients with metabolic disorders, the use of medications such as antibiotics and psychiatric drugs may further prolong the QT interval, increasing risk.

Recent evidence also suggests that insulin resistance, a key component of cardiometabolic disease, is independently associated with QT interval prolongation [6]. However, current clinical guidelines for diabetes, obesity, and related disorders do not include QTc monitoring as a routine risk assessment tool.

## Evidence Base

This policy brief is based on:

• Iranian cohort studies involving 7,071 participants followed over three years [2][3]

• A meta-analysis demonstrating QT prolongation associated with insulin resistance [6]

Key findings include:

• Anemia: OR=1.6 (95%CI:1.12-2.28) [4]

• MAFLD: OR=1.47 (95%CI:1.18-1.84, P<0.001) [3]

• Insulin resistance: WMD=12.38 (95%CI:5.51-19.25) [6]

• Obesity: significant QTc prolongation (P<0.001) [5]

• Prolonged QTc: OR=2.98 (95%CI:1.16-7.66, P=0.023) [2]

These findings indicate that QTc prolongation is a clinically significant intermediary linking metabolic disorders to increased cardiovascular risk.

## Policy Options

**Table:** Table1. Clinical Implementation Protocol for QTc Monitoring in Patients with Metabolic Disorders

Component	Recommendation	Target Population	Frequency	Clinical Action
Baseline ECG	12-lead ECG with QTc calculation	All patients	At diagnosis	Risk stratification
Annual Monitoring	Repeat ECG	All patients	Annually	Early detection
Pre-medication Screening	ECG before QT-prolonging drugs	At-risk patients	Before therapy	Prevent drug-induced QT prolongation
During Therapy	Follow-up ECG	Treated patients	As needed	Adjust therapy
High-risk Monitoring	Intensive ECG follow-up	High-risk patients	Every 3-6 months	Prevent arrhythmias
Abnormal QTc	Advanced evaluation	Patients with prolonged QTc	As needed	Specialist referral

ECG: Electrocardiogram; QTc: Corrected QT Interval

Option 1: Short-Term (6 Months)

• Implement QTc alerts in electronic health systems

• Develop temporary clinical guidelines

Advantages: Rapid implementation, low cost

Limitations: Limited coverage

Option 2: Medium-Term (12-24 Months)

• Pilot implementation in selected provinces

• Conduct clinical and economic evaluation

Advantages: Generates local evidence

Limitations: Requires coordination

Option 3: Long-Term Program (2 Years)

• Establish national ECG/QTc registry

• Develop standardized physician training

• Expand diagnostic capacity

Advantages: Sustainable population impact

Limitations: Higher cost and longer timeline

Option 4: Optimal Strategy (Recommended)

Implementation of a national QTc monitoring protocol, including:

• Baseline ECG at diagnosis

• Annual ECG monitoring

• Screening before and during QT-prolonging drug therapy

• Physician training programs

• Insurance coverage

Advantages: High effectiveness, scalable

Limitations: Requires national coordination

Table 1 presents the clinical implementation protocol for QTC monitoring.

## Cost-Effectiveness Considerations

**Table:** Table2. Cost-Effectiveness Analysis of QTc Monitoring Strategies

Strategy	Cost per Patient (USD)	Clinical Benefit	Economic Impact
No screening	0	Late detection	High cost burden
Baseline ECG	<1	Initial stratification	Minimal benefit
Annual ECG	1-2	Early detection	Cost-effective
Targeted monitoring	2-5	High-risk prevention	Highly cost-effective
Comprehensive program	3-10	Continuous prevention	Very high value
Cardiac event treatment	10,000-15,000	Acute care	Very high cost

$ : US dollar

Electrocardiography is a low-cost and widely available diagnostic tool. In Iran, the cost of a single ECG is less than one dollar, whereas treatment of acute cardiac events such as myocardial infarction may cost between $10,000 and $15,000 per patient.(table 2)

Routine QTc monitoring can:

• Reduce hospitalizations and emergency care costs

• Prevent premature mortality

Improve allocation of healthcare resources

Given the strong association between QTc prolongation and adverse outcomes [2][3][4][5][6], even modest reductions in cardiac events would offset implementation costs.

## Policy Implications

Implementation of QTc monitoring is expected to:

• Enable early identification of high-risk individuals

• Reduce sudden cardiac death and premature mortality

• Decrease healthcare expenditures related to acute cardiac events

• Improve equity in access to preventive services

• Strengthen cardiovascular risk prediction models

• Inform updates to clinical guidelines

## Implementation Considerations

• Successful implementation requires:

• Integration into existing primary healthcare systems

• Training of physicians in ECG interpretation

• Development of electronic alert systems

• Expansion of insurance coverage

• Continuous monitoring and evaluation

A phased implementation strategy is recommended.

## Risks and Mitigation

Potential risks include:

• Increased workload for healthcare providers

• Variability in ECG quality and interpretation

• Short-term increase in healthcare costs

• Risk of overdiagnosis

• Geographic disparities in access

These risks can be mitigated through standardized training, system integration, and alignment with healthcare financing mechanisms. Importantly, the risk of inaction continued preventable sudden cardiac deaths remains substantially greater.

## Conclusion and Key Recommendation

Metabolic disorders significantly increase the risk of QTc prolongation and sudden cardiac death. Evidence from cohort studies and meta-analyses supports routine ECG-based QTc monitoring as a cost-effective and scalable intervention [2][3][4][5][6].

## Key Recommendation:

Adopt a national QTc monitoring program incorporating baseline and annual ECG assessments as part of standard care for patients with metabolic disorders.

## Conflict of Interest

The authors declare no conflict of interest.

## AI Disclosure Statement

During the preparation of this manuscript, the authors used ChatGPT.com for language editing, grammar improvement, and liboberry.com for reference management. After its use, the authors thoroughly reviewed, verified, and revised all AI-assisted content to ensure accuracy and originality. The authors take full responsibility for the integrity and final content of the published article.
